# Cholera toxin B scaffolded, focused SIV V2 epitope elicits antibodies that influence the risk of SIV_mac251_ acquisition in macaques

**DOI:** 10.3389/fimmu.2023.1139402

**Published:** 2023-04-21

**Authors:** Mohammad Arif Rahman, Manuel Becerra-Flores, Yury Patskovsky, Isabela Silva de Castro, Massimiliano Bissa, Shraddha Basu, Xiaoying Shen, LaTonya D. Williams, Sarkis Sarkis, Kombo F. N’guessan, Celia LaBranche, Georgia D. Tomaras, Pyone Pyone Aye, Ronald Veazey, Dominic Paquin-Proulx, Mangala Rao, Genoveffa Franchini, Timothy Cardozo

**Affiliations:** ^1^ Animal Models and Retroviral Vaccines Section, National Cancer Institute, NIH Bethesda, MD, United States; ^2^ NYU Langone Health, New York University School of Medicine, New York, NY, United States; ^3^ United States Military HIV Research Program, Walter Reed Army Institute of Research, Silver Spring, MD, United States; ^4^ Henry M. Jackson Foundation for the Advancement of Military Medicine, Inc., Bethesda, MD, United States; ^5^ Department of Surgery, Duke University School of Medicine, Durham, NC, United States; ^6^ Duke Human Vaccine Institute, Duke University School of Medicine, Durham, NC, United States; ^7^ Veterinary Medicine, Tulane National Primate Research Center, Covington, LA, United States; ^8^ Division of Comparative Pathology, Department of Pathology and Laboratory Medicine, Tulane National Primate Research Center, Tulane University School of Medicine, Covington, LA, United States

**Keywords:** SIV, single epitope subunit vaccine, cholera toxin B scaffold vaccine, DNA/ALVAC vaccine, ADCC, efferocytosis, CCR5^-^ α_4_β_7_^-^ CD4^+^ T cells

## Abstract

**Introduction:**

An efficacious HIV vaccine will need to elicit a complex package of innate, humoral, and cellular immune responses. This complex package of responses to vaccine candidates has been studied and yielded important results, yet it has been a recurring challenge to determine the magnitude and protective effect of specific *in vivo* immune responses in isolation. We therefore designed a single, viral-spike-apical, epitope-focused V2 loop immunogen to reveal individual vaccine-elicited immune factors that contribute to protection against HIV/SIV.

**Method:**

We generated a novel vaccine by incorporating the V2 loop B-cell epitope in the cholera toxin B (CTB) scaffold and compared two new immunization regimens to a historically protective ‘standard’ vaccine regimen (SVR) consisting of 2xDNA prime boosted with 2xALVAC-SIV and 1xΔV1gp120. We immunized a cohort of macaques with 5xCTB-V2c vaccine+alum intramuscularly simultaneously with topical intrarectal vaccination of CTB-V2c vaccine without alum (5xCTB-V2/alum). In a second group, we tested a modified version of the SVR consisting of 2xDNA prime and boosted with 1xALVAC-SIV and 2xALVAC-SIV+CTB-V2/alum, (DA/CTB-V2c/alum).

**Results:**

In the absence of any other anti-viral antibodies, V2c epitope was highly immunogenic when incorporated in the CTB scaffold and generated highly functional anti-V2c antibodies in the vaccinated animals. 5xCTB-V2c/alum vaccination mediated non-neutralizing ADCC activity and efferocytosis, but produced low avidity, trogocytosis, and no neutralization of tier 1 virus. Furthermore, DA/CTB-V2c/alum vaccination also generated lower total ADCC activity, avidity, and neutralization compared to the SVR. These data suggest that the ΔV1gp120 boost in the SVR yielded more favorable immune responses than its CTB-V2c counterpart. Vaccination with the SVR generates CCR5^-^ α4β7^+^CD4^+^ Th1, Th2, and Th17 cells, which are less likely to be infected by SIV/HIV and likely contributed to the protection afforded in this regimen. The 5xCTB-V2c/alum regimen likewise elicited higher circulating CCR5^-^ α4β7^+^ CD4^+^ T cells and mucosal α4β7^+^ CD4^+^ T cells compared to the DA/CTB-V2c/alum regimen, whereas the first cell type was associated with reduced risk of viral acquisition.

**Conclusion:**

Taken together, these data suggest that individual viral spike B-cell epitopes can be highly immunogenic and functional as isolated immunogens, although they might not be sufficient on their own to provide full protection against HIV/SIV infection.

## 1 Introduction

Efficacious subunit vaccines, whose protective immunologic profile is typically directly associated with anti-viral antibodies (Abs), are commonly licensed for immunization against viral pathogens (1). The development of vaccines against enveloped RNA viruses such as HIV, SARS-Cov-2, RSV, and influenza has generally been less successful. Linear B-cell epitopes at the apex of these viral spikes elicit Abs that mediate neutralization, antibody-dependent cellular cytotoxicity (ADCC), and phagocytosis (ADCP) and complement fixation, which might be important for protection against viral infection (1, 2). The success of the recent COVID-19 vaccine is a notable exception in the development of an RNA vaccine. In this platform, vaccination elicits antibodies that proactively target the apex of the prefusion conformation of the trimeric viral spike, inhibiting virion formation and effectively preventing pathogenesis/disease progression (3–8).

In the case of HIV, only one of the nine clinical trials conducted so far has afforded significant protection from infection (9–18). The prime-boost vaccine regimen tested in the RV144 phase III clinical trial achieved a modest 31.2% decrease in the risk of clinical HIV acquisition sustained 3 years after immunization with an ALVAC-HIV prime and boosted with ALVAC-HIV and gp120 formulated in alum (14, 19). The primary correlate of reduced risk in RV144 was the level of IgG binding to the V1/V2 variable loops of gp120 scaffolded on gp70 (20). Notably, neutralizing antibodies were not associated with protection and had the highest odds ratio for viral acquisition (20). In vaccinees with low IgA levels, ADCC was a secondary correlate of reduced risk of HIV acquisition (20).

The vaccine efficacy of the RV144 trial was recapitulated and confirmed in two independent studies in the macaque model using the same vaccine modalities based on SIV immunogens. In addition to confirming the findings of RV144, the investigations revealed the association of anti-V2 IgG levels with a decreased risk of acquisition following intrarectal exposure to SIV_mac251_ (21, 22). The V2 B-cell epitopes (20, 22–25) targeted by these Abs rest near the site of gp120 binding to the host α_4_β_7_ integrin receptor (26) at the apex of the HIV/SIV trimeric viral spike. Additional macaque studies have optimized the RV144 vaccine and produced a more efficacious (52%) regimen (27) wherein the prime was substituted with DNA and the V1 envelope region was deleted (ΔV1) in order to expose protective epitopes at the apex of the trimeric viral spike (27–29), referred to here as standard vaccine regimen (SVR) of 2xΔV1 DNAgp160+p57 Gag and boosted with ALVAC-SIV and ALVAC-SIV/ΔV1 gp120/alum. The SVR demonstrated that antibodies recognizing these specific, spike-apical, linear B-cell epitopes bind to virions and mediate ADCC associated with a decreased risk of virus acquisition (27–29). In these same studies, anti-V2 polyclonal responses to SIV peptide probes encompassing position 169 and 181 of V2 were also associated with a transient decrease in virus burden in plasma and mucosal tissues in vaccinated animals that became infected. The decreased risk of SIV_mac251_ acquisition was not only associated with V2-specific antibodies and V2-specific ADCC, but also with mucosal envelope specific NKp44^+^ cells producing IL-17, CD14^+^ monocytes mediating efferocytosis, and Th1/Th2 cells expressing no or low levels of CCR5 (21, 27–31)

Here, we generated a novel vaccine platform, CTB-V2c, by scaffolding a mucosal-targeting, pentameric particle approaching the size of a typical virus-like particle (VLP) from cholera toxin B (CTB) onto the SIV V2c B-cell epitope, favoring an α-helical conformation of V2. We investigated the preliminary immunogenicity of CTB-V2c in rabbits and generated two immunization regimens to test in macaques. In the first, we generated a vaccine using CTB-V2c as a standalone immunogen (5xCTB-V2/alum). Additionally, we assessed the immunogenicity of a modified version of the SVR, with 2xDNAprimes and boosted with 1xALVAC-SIV and 2xALVAC-SIV+CTB-V2c/alum (DA/CTB-V2c/alum). Animals were challenged with SIV_mac251_ in order to reveal correlations between specific vaccine-elicited humoral and cellular immune factors and per-challenge risk of viral acquisition.

## 2 Materials and methods

### 2.1 Animals

#### 2.1.1 Rabbits

Six, 3-month-old New Zealand White female rabbits of 2.6–3 kg, bred locally at Pocono Rabbit Farms and Laboratory (PRFL, Canadensis, PA) were housed individually and kept under observation during an acclimatization period of two weeks. Animals were randomly allocated into two groups (4 rabbits in group 1, 2 rabbits in group 2): group 1 was immunized with 200 μg CTB-SIV_sm543_-V2c and group 2 with 200 μg CTB-SIV_mac251_-V2c. The immunization schedule consisted of two immunizations over a 2-week interval, with the first denoted as week 0. In order to assess the humoral immune response (IgG levels), blood samples were collected from the marginal ear vein prior to each immunization and every two weeks thereafter until week 10. Serum samples were obtained from blood and stored at -20 C until use. All animal work was performed on location at PRFL under PRFL IACUC approval PRF2A, with umbrella approval by the NYU Langone Health IACUC committee.

#### 2.1.2 Macaques

Fifteen colony-bred, male, Indian rhesus macaques (Macaca mulatta) obtained from Covance Research Products (Alice, TX) were used in these studies. Animals were housed at the Tulane University National Primate Research Center. All animals were handled in accordance with the standards of the Association for the Assessment and Accreditation of Laboratory Animal Care (AAALAC) standards in an AAALAC-accredited facility (OLAW, Animal Welfare Assurance). All animal care and procedures were carried out under protocols approved by Tulane Animal Care and Use Committees. Animals were closely monitored daily for any signs of illness, and appropriate medical care was provided as needed. Animals were socially housed per the approved ACUC protocol and social compatibility except during the viral challenge phase when they were individually housed.

Animals were closely monitored daily for any signs of illness, and appropriate medical care was provided as needed. Animals were socially housed per the approved ACUC protocol and social compatibility except during the viral challenge phase when they were individually housed. All clinical procedures, including biopsy collection, administration of anesthetics and analgesics, and euthanasia, were carried out under the direction of a laboratory animal veterinarian. Steps were taken to ensure the welfare of the animals and minimize discomfort of all animals used in this study. Animals were fed daily with a fresh diet of primate biscuits, fruit, peanuts, and other food items to maintain body weight or normal growth. Animals were monitored for psychological well-being and provided with physical enrichment including sanitized toys, destructible enrichment (cardboard and other paper products), and audio and visual stimulation.

### 2.2 Vaccine

#### 2.2.1 Immunogen epitope probe peptide design and mutational analysis

The amino acid sequence of the V2 loop of SIV_smE5433_ from positions 167 to position 180 (V2c; HXBC2 numbering) was previously identified as a linear B-cell epitope correlated with protection in the low dose, multiple challenge SIV_mac251_ macaque animal model (27). Briefly, two peptides, one bearing SIV_smE5433_ sequence DKKIEYNETWYSRD and the other bearing SIV_mac251_ sequence DKTKEYNETWYSTD, were designed to favor an α-helix structure by NMR-validated, *ab initio* computational folding (32–35). These peptide sequences were synthesized as probes suitable for ELISA by adding an N-terminal biotin and tri-glycine linker (biotin-GGG-V2c sequence; Genemed Inc., San Francisco, CA). The point mutants described in the text were synthesized commercially.

#### 2.2.2 Protein immunogen production

The cDNA for recombinant CTB (~102 amino acids in length) was fused in frame to a periplasmic-targeting (SEC translocon), signal peptide at the N-terminus and to the SIV_smE5433_ V2c linear B-cell epitope at the C-terminus, respectively, to form CTB-V2c^SIV^ and cloned into a pET32a expression vector. After transforming *Escherichia coli* BL21 (DE3), expression of the recombinant protein was induced with IPTG. Cells were collected and the periplasmic fraction (signal peptide cleaved during translocation to periplasm) was isolated using the standard osmotic shock protocol. Since correctly folded CTB binds galactose in the same way as mucosal GM1-gangliosides, CTB-V2c^SIV^ was isolated by affinity chromatography on galactose-agarose beads (Thermo Fisher Scientific, Waltham, MA), which purifies only pentameric, GM1-ganglioside-binding, mucosal targeting CTB-V2c^SIV^ and removes all endotoxin. CTB-V2c^SIV^ was then further purified by gel-filtration using Supplementaryerdex S200 (GE Bioscience*s*, Niskayuna, NY). The expression and purification protocol were optimized to produce over 20 mg of greater than 99% pure CTB-V2c^SIV^ per 1 liter of bacterial culture.

#### 2.2.3 Analytical validation and authentication of CTB-V2c^SIV^

Recombinant CTB-V2c^SIV^ was validated and authenticated using SDS-PAGE and mass-spectrometry, and by crystallization and solving the crystal structure (2.1 Å). The ESI MS method was used to confirm the total protein mass. The MS/MS spectrum was generated by fragmenting the 12+charge state from the full MS spectrum with electron transfer dissociation (EDT) and using the 20 *ms* ETD activation and 20% supplemental activation, respectively. This caused the protein backbone to dissociate into *b, c, y* and *z*-type fragment ions, which were used to confirm the correct protein sequence and the presence of a disulfide bond. Pure CTB-V2c^SIV^ protein was concentrated to 20 mg/ml, mixed with galactose and crystallized using MCSG crystallization screens and then the crystals were further optimized. The high-resolution X-ray diffraction data from the needle-like protein crystals frozen in liquid nitrogen were collected at the micro-focus AMX beamline 17-ID (National Synchrotron Light Source, Brookhaven National Laboratory, Upton, NY). The data were processed by *XDS*. The crystal structure was solved by molecular replacement and refined using *REFMAC* and *COOT* software. The 2.1 Å crystal structure was refined to the R_factor_ of ~18% and R_free_ of 22.2%, respectively. The crystal structure confirmed the pentameric composition of CTB-V2c^SIV^ and the existence of a properly folded GM1-receptor (galactose) binding site (one per each monomer or 5 per each pentamer), which was detected by the presence of tightly bound galactose. The C-terminal epitope was also partially observed in electron density maps but is expected to be flexible for maximal immunogenicity. Since the mucosal receptor binding site and the inserted epitope are located at the opposite sides of the pentamer, CTB binding to the GM1 receptor is unlikely to interfere with the epitope exposure. The protein is endotoxin-free due to purification with the galactose column (the recommended level for toxoid vaccines is nevertheless <200,000 EU/ml (36)). Thus, a pharmaceutical grade CTB-V2c^SIV^ synthetic fusion immunogen was produced in scalable milligram amounts. This protein was formulated 1:1 with aluminum hydroxide Alhydrogel (Invivogen, San Diego, CA) as previously described (28) for IM immunization and in PBS for topical intrarectal inoculation.

### 2.3 Vaccination and challenge study in macaques

Fifteen male Indian rhesus macaques aged 5 to 6 years at study initiation, and negative for SIV, simian retrovirus (SRV), and Simian T-cell leukemia viruses (STLVs) were used in this study. Macaques were randomized into groups based on age and weight. Immunological data from an additional twenty-four male and female historical macaques were also used in this study (27, 31).

#### 2.3.1 5xCTB-V2c/alum vaccine régimen

Five macaques were immunized with 500 μg of CTB-V2c^SIV^ protein formulated 1:1 in 500 μg alum IM and 1 mg CTB-V2c^SIV^ protein in PBS intrarectally (IR) at weeks 0, 4, 8, 13 and 40 weeks.

#### 2.3.2 DA/CTB-V2c/alum vaccine régimen

Five additional macaques were immunized twice with DNA-SIV intramuscularly at weeks 0 and 4 as previously described (28). Each vaccination contained a total of 4 mg of DNA in 1.2 mL PBS. The animals were given the following DNA constructs: 206S p57Gag (1 mg), 209S MCP3p39 gag (1 mg), ΔV1 _M766_gp160 (2 mg). At weeks 8, 13, and 40, the five DNA-immunized macaques were boosted with intramuscular inoculations of 10^8^ Plaque Forming Units (PFU) of recombinant ALVAC (vCP2432), expressing SIV_mac251_ *gag-pro* and *gp120TM* (Sanofi Pasteur, Bridgewater, NJ). At week 13 and 40, the five DA vaccinated macaques also received 500 μg of CTB-V2c^SIV^ protein formulated in alum IM and 1 mg of CTB-V2c^SIV^ protein in PBS intrarectally (IR).

#### 2.3.3 Standard vaccine regimen

Twelve male and two female macaques were immunized as previously described (27). Briefly, macaques were immunized twice intramuscularly at weeks 0 and 4 with DNA-SIV expressing gag and ΔV1 gp160_M766_. At weeks 8 and 12 the animals were boosted with intramuscular inoculations of 10^8^ Plaque Forming Units (PFU) of recombinant ALVAC (vCP2432). At week 12 the animals also received a ΔV1 gp120+alum SIV_mac251_ gp120_M766_ monovalent boost.

#### 2.3.4 Alternate SVR using bivalent SIV gp120 proteins

Ten female macaques were immunized as previously described [Study 1 (31)]. Briefly, macaques were immunized twice intramuscularly at weeks 0 and 4 with DNA-SIV expressing gag and _M766_gp160. At weeks 8 and 12 the animals were boosted with intramuscular inoculations of 10^8^ PFUs of recombinant ALVAC (vCP2432). At week 12, macaques also received 200 μg each of SIV_mac251-M766_ and SIV_smE660-CG7V_ gp120-gD proteins formulated in alum Alhydrogel (Invivogen, San Diego, CA). The animals were not challenged with SIV_mac251_.

#### 2.3.5 SIV_mac251_ challenge study

All 10 5xCTB-V2c/alum and DA/CTB-V2c/alum vaccinated macaques and 5 naïve control macaques were challenged intrarectally with 11 repeated, low doses of pathogenic SIV_mac251_ once a week. The stock of SIV_mac251_ was propagated in macaque cells (QBI#305342b, Quality Biological, Gaithersburg, MD). Challenge was initiated at 4 weeks following the last immunization (week 44 for immunized animals) and each animal was intrarectally administered 1 mL of SIV_mac251_ diluted in RPMI 1640 (Gibco, Waltham, MA) to a final concentration of 400 TCID_50_/mL (evaluated in rhesus 221 cells).

Twelve male and two female macaques immunized with the standard vaccine regimen were exposed intrarectally to 11 repeated weekly doses of SIV_mac251_ 5 weeks post last immunization (Week 17). Animals were not challenged further after becoming PCR positive.

### 2.4 Viral RNA and DNA and CD4^+^ T cell count

The RNA copies of SIV_mac251_ in plasma were quantified by nucleic acid sequence-based amplification as previously described (28). CD20^+^, CD4^+^, and CD8^+^ T cell counts in whole blood were assessed by flow cytometry using a previously described protocol (28).

### 2.5 Binding of serum Abs to antigens

ELISA assays for reactivity of macaque and rabbit serum with biotinylated V2c, V2b and V1a peptides were performed as previously described (35, 37). Briefly, streptavidin coated plates were incubated at room temperature for 3 hours in wash buffer (Tris buffered saline containing 0.1% BSA and 0.05% Tween 20) with the biotinylated peptides or gp120 variants (WT, ΔV1) at 100 ng/well, followed by an overnight incubation at 4˚C with serially diluted serum samples in duplicate, or 1µg/ml mAbs, in 100 µL/well of wash buffer. Plates were incubated at room temperature for 2 hours with goat, anti-monkey IgG for macaque serum and goat, anti-rabbit IgG, both conjugated with alkaline phosphatase, at 0.5 μg/mL in 100 μL/well of wash buffer. Plates were incubated with alkaline phosphatase substrate in developing buffer (PBS, 1M DEA, 0.24M MgCl2.6H2O, pH 9.8) and read at OD 405 nm at 30 minutes.

### 2.6 Binding antibody multiplex assay

Week 14 serum IgG binding antibody responses were evaluated against SIV peptides by binding antibody multiplex assay (BAMA) as previously described (20, 23, 38, 39). A panel of five biotinylated SIV peptides (SIV V2-long, SIV_V2b_164, SIV_V1_543, SIV_V2c_251, SIV_V2c_543, SIVmac239_V2b_166) were conjugated to NeutrAvidin-coupled magnetic beads and mixed with serum samples tested at a 1:40 dilution, assayed in duplicate. IgG binding was detected using mouse anti-human IgG-Fc PE (Southern Biotech, Brimingham, AL). Beads were analyzed on a Luminex^®^ FLEXMAP 3D^®^ instrument, with binding magnitude expressed as mean fluorescence intensity (MFI). IgG purified from a SIV_mac251_-infected rhesus macaque was included as an assay positive control and blank (uncoupled) beads as a negative control. A response was considered positive if the MFI was (1) greater than 100, (2) greater than the antigen-specific cutoff (95^th^ percentile of all pre-immunization sample binding to the antigen), and (3) 3-fold higher than the matched pre-immunization sample before and after blank bead subtraction.

### 2.7 Antibody dependent cellular cytotoxicity assay

#### 2.7.1 CEM-based assay

ADCC activity was assessed as previously described using EGFP-CEM-NKr-CCR5-SNAP cells that constitutively express GFP as targets (30, 40). Briefly, one million target cells were incubated with 50 μg of wild type or ΔV1 gp120 protein for 2 h at 37°C. The coated target cells were washed and labeled with SNAP-Surface^®^ Alexa Fluor^®^ 647 (New England Biolabs, Connecticut, USA) per manufacturer recommendations for 30 min at RT. Plasma samples, heat inactivated at 56°C for 30 min, were serially diluted (7 ten-fold dilutions starting at 1:10) and 100 μl were added to wells of a 96-well V-bottom plate (Millipore Sigma, Burlington, MA). 5000 target cells (50 μl) and 250,000 human PBMCs (50 μl) were added as effectors to each well to give an effector/target (E/T) ratio of 50:1. The plate was incubated at 37°C for 2 h followed by two PBS washes. The cells were resuspended in 200 μl of a 1% PBS–paraformaldehyde solution and acquired on an LSRII equipped with a high throughput system (BD Biosciences, San Jose, CA). Specific killing was measured by loss of GFP from the SNAP-Alexa647+ target cells. Target and effector cells cultured in the presence of R10 medium were used as background. Anti-SIVmac gp120 monoclonal antibody KK17 (NIH AIDS reagent program) was used as a positive control. Normalized percent killing was calculated as: (killing in the presence of plasma – background)/(killing in the positive control- background) X100. The ADCC endpoint titer is defined as the reciprocal dilution at which the percent ADCC killing was greater than the mean percent killing of the background wells containing medium only with target and effector cells, plus three standard deviations.

#### 2.7.2 V2-specific ADCC killing by CEM-based assay

F(ab’)2 fragments were prepared from NCI05 or NCI09 mAb, as these antibodies recognize overlapping conformationally distinct V2 epitopes (27), using Pierce F(ab’)_2_ Micro Preparation Kit (Thermo Fisher Scientific) following the manufacturer’s instructions. SDS-page gel with the recovered F(ab’)_2_ was run and silver stained (Silver Quest staining Kit, Invitrogen) according to the manufacturer’s instructions, to assure the purity of the F(ab’)2 fragments. Target cells, coated with gp120 as indicated and labeled with SNAP-Surface^®^ Alexa Fluor^®^ 647, were incubated for 1 h at 37°C with 5 μg/ml of purified F(ab’)_2_ fragments from NCI05 or NCI09 monoclonal antibodies. Cells incubated without F(ab’)_2_ were also used to determine total ADCC killing. These target cells were subsequently used in the ADCC assay as described above (27, 29, 31). V2 specific ADCC killing was determined by subtracting the ADCC killing in the presence of F(ab’)2 from total ADCC killing of the respective samples.

### 2.8 Surface plasmon resonance (Biacore)

Antibody avidity determinations were conducted using the Biacore 4000 surface plasmon resonance (SPR) system as previously described (41–44). Briefly, the immobilizations were performed using a standard amine-coupling kit. The CM-5 sensor chip surface was activated with a 1:1 mixture of 0.4 M 1-ethyl-3-(3-dimethylaminopropyl) carbodiimide hydrochloride (EDC, Cytiva) and 0.1 M N-hydroxysuccinimide (NHS, Cytiva) for 600 sec. Proteins CTB-V2C (10 μg/ml; spots 1 and 2 of flow cell 2) or (4 μg/ml in spots 4 and 5 of flow cell 2); M766gp120 (10 μg/ml; spots 1 and 2 of flow cell 3) or (4 μg/ml in spots 4 and 5 of flow cell 3), and dV1gp120 (10 μg/ml; spots 1 and 2 of flow cell 4) or (4 μg/ml in spots 4 and 5 of flow cell 4), in 10mM sodium acetate pH 4.5 were immobilized to spots 1 and 2, 4, and 5 of the CM5 sensor chip resulting in 6124-4502 RU (high density) and 379-510 RU (low density) for CTB-V2C; 2686-2552 RU (high density) and 409-278 RU (low density) for M766gp120; and 4244-4206 RU (high density) and 704-616 RU (low density) for dV1gp120, respectively for the flow cells 2, 3, and 4. Spot 3 in each flow cell was left unmodified to serve as a reference. Flow cell 1 served as the blank. The immobilized surface was then deactivated with 1.0 M ethanolamine-HCl pH 8.5 for 600s. Following the surface preparation, heat-inactivated (56°C) plasma samples were diluted 1:50 in running buffer (10 mM Hepes, 150 mM NaCl, 0.005% Tween-20, pH7.4) and injected onto the protein immobilized surface for 240s-250s followed by dissociation for 900-1300s. Four replicates for each sample were collected at rate of 10 Hz, with an analysis temperature of 25°C. All sample injections were conducted at a flow rate of 10 μL/min. The bound surface was regenerated with 150 mM HCl for 60s. Data analysis was performed using Biacore 4000 Evaluation software 4.1 with double subtractions for the unmodified surface and buffer blank. Fitting was conducted using the dissociation mode integrated with Evaluation software 4.1. The data are shown as avidity score, which was calculated as Response Unit/K_d_.

### 2.9 Trogocytosis

Trogocytosis was measured using a previously described assay (45). CEM.NKR.CCR5 cells were washed with PBS and stained with PKH26 (Sigma-Aldrich, St-Louis, MO, USA) at 2μM in Diluent C at RT for 5 min. Cells were then washed with R-10, resuspended in R-10, and incubated with WT gp120 or ΔV1 gp120 for 1 h at RT in 96-well polypropylene plates. Cells were washed twice with R-10 and incubated with 300-fold diluted plasma samples. Cryopreserved healthy control PBMC were next added in R-10 at an effector to target (E:T) cell ratio of 50:1 and then incubated for 5 h at 37°C. After the incubation, cells were washed, stained with live/dead aqua fixable stain and anti-CD14 APC-H7 (clone MΦP9, BD, San Jose, CA, USA), washed again, and fixed with 4% formaldehyde (Tousimis, Rockville, MD). Fluorescence was evaluated on an LSRII flow cytometer (BD Biosciences). Trogocytosis was evaluated by measuring the PKH26 mean fluorescence intensity of the live CD14^+^ cells.

### 2.10 Serum neutralizing antibodies

Neutralization in the serum of vaccinated animals was measured as a reduction in luciferase reporter gene expression after a single round of infection in TZM-b1 cells as described previously (46, 47). TZM-bl cells were obtained from the NIH AIDS Research and Reference Reagent Program, contributed by John Kappes and Xiaoyun Wu. Test samples were serial diluted (3-fold dilution in duplicate) and incubated with 200 TCID_50_ of SIV_mac251.6_ (ID #1636DB2) in a total volume of 150 μl for 1 h at 37°C in 96-well flat-bottom culture plates. TZM-bl cells were trypsinized and added to each well (10,000 cells in 100 μl of growth medium containing 20 μg/mL DEAE dextran). A set of wells with cells and virus was used as virus control, and another set of wells with cells only was used as background control. After 48 h incubation, the cells were lysed by the addition of Britelite (PerkinElmer Life Sciences, Waltham, MA), and three quarters of the cell lysate were transferred to a 96-well black solid plate (Corning Costar, Tewksbury, Massachusetts) for luminescence measurement. Neutralization titers are defined as the dilution at which relative luminescence units were reduced by 50% or 80% compared to that in virus control wells after subtraction of background relative luminescence units.

### 2.11 Efferocytosis assay

The frequency of efferocytotic CD14^+^ cells was assessed by Efferocytosis Assay kit (#601770, Cayman Chemical company, Ann Arbor, MI). CD14^+^ cells were used as effector cells, whereas apoptotic neutrophils were used as target cells. The protocol was readapted in order to use CD14^+^ monocyte cells rather than differentiated macrophages due to the low cell availability (29, 31). CD14^+^ cells were isolated from cryopreserved PBMCs (10x10^6^ cells) collected following pre-immunization and 2 weeks post last immunization (week 14) or 5 weeks post last immunization (week 17) by using non-human primate CD14 MicroBeads (#130-091-097, Miltenyi Biotec Inc.) and following manufacturer instructions. At the end of the separation cells were counted and stained with CytoTell Blue provided in the kit and following manufacturer instructions. One unrelated macaque was used as source of neutrophils as target cells. Neutrophils were isolated as previously described (48). Briefly, following isolation of PBMCs by Ficoll Plaque (GE Healthcare), the cellular pellet was added to an equal volume of 20% dextran in water, gently mixed, and incubated for 1 min. Approximately three volumes of PBS were added, mixed again and incubated in the dark for 50-60 minutes. At the end of incubation, the clear layer at the top of the tube containing neutrophils was collected. Cells were pelleted and treated with ACK lysing buffer (Quality Biological, Gaithersburg, MD, USA) for 5 min at 37°C, washed with R10 and counted. Neutrophils were stained with CFSE provided in the kit and following manufacturer instructions. Apoptosis of neutrophils was induced by treatment with Staurosporine Apoptosis inducer provided in the kit. Briefly, isolated cells were resuspended in R10 containing Staurosporine diluted 1:1000 and incubated at 37°C for 3 hours. At the end of the incubation cells were washed twice with R10 and used for the efferocytosis assay. Subsequently, effector and apoptotic target cells were cultured alone (as controls) or cocultured at a ratio of one effector CD14^+^ cell and three target apoptotic neutrophils. Cells were incubated at 37°C for 12 hours. At the end of the coculture, cells were washed with PBS, fixed with 1% paraformaldehyde in PBS and acquired on a FACSymphony A5 and examined using FACSDiva software (BD Biosciences) by acquiring all stained cells. Data were further analyzed using FlowJo v10.1 (BD Biosciences). The frequency of efferocytotic CD14^+^ cells was determined as the frequency of double-positive cells for CytoTell Blue and CFSE on the CytoTell Blue positive monocytes.

### 2.12 Flow cytometry analysis

#### 2.12.1 Blood T cell panel

To assess blood T cell subsets cellular correlates of protection, which were identified in previous studies using the DA/Env platform (27, 28), cryopreserved PBMCs (5-10 x 10^6^ cells) collected at baseline, 2 weeks post 4^th^ immunization (Week 15) and 2 weeks post 5^th^ immunization (Week 42) were thawed, cells were stained for live cells with Live/Dead Blue dye (cat. #L34962, 0.5 μl) from Thermo Fisher Scientific; followed by surface staining with the following: PE-Cy5 anti-CD95 (clone DX2; cat. #15-0959-42, 5.0 µl), APC-Cy7 anti-CD11b (clone ICRF44; cat. #47-0118-42, 5.0 µl) from Thermo Fisher Scientific; Alexa 700 anti-CD20 (clone 2H7; cat. #560631, 5.0 µl), BV650 anti-CCR5 (clone 3A9; cat. #564999, 5.0 µl), BV711 anti-CD4 (clone L200; cat. #563913, 5.0 µl), BV786 anti-CD45 (clone D058-1283; cat. #563861, 5.0 µl), BUV496 anti-CD16 (clone 3G8; cat. #612944, 5.0 µl), BUV737 anti-CD3 (clone SP34-2; cat. #741872, 5.0 µl), from BD Biosciences; BV605 anti-CCR6 (clone G034E3; cat. #353420, 5.0 µl), BV750 anti-CXCR3 (clone G025H7; cat. #353722, 5.0 µl) from Biolegend and APC anti-a4b7 (clone A4B7R1; cat. #051514AB, 5.0 µl) from NHP Reagent Resource for 30 minutes at room temperature. This was followed by permeabilization with a FOX3-transcription buffer set (cat. #00-5523-00) from eBioscience according to the manufacture’s recommendation and subsequently intracellular staining with the following: BV510 anti- Ki67 (clone B56; cat. #563462, 5.0 µl) and PerCP-Cy5.5 anti- FoxP3 (clone 236A/E7; cat. #561493, 5.0 µl) from BD Biosciences for 30 minutes at room temperature. Flow cytometry acquisitions were performed on a FACSymphony A5 and examined using FACSDiva software (BD Biosciences).

#### 2.12.2 Rectal T cell panel

The frequency T cells were measured in macaque rectal mucosa pre vaccination and 2-week post 4^th^ vaccination (week 15). and 1 week post last vaccination (week 41). Freshly collected rectal biopsies were digested with collagenase (2 mg/ml; Sigma-Aldrich) in the absence of FBS in 37°C for 1 hour, then it was mechanically separated by using a 10 ml syringe with a blunt head canula. It was washed with R10 and pass through 70 µm cell strainer. Single cells were counted and used for the experiment. Two million cells were phenotyped by staining with Live/Dead Aqua Dye (cat. #L34966, 0.5 µl) from Thermo Fisher, followed by surface staining with the following: APC-Cy7 anti-CD11b (clone ICRF44; cat. #47-0118-42, 5.0 µl) from Thermo Fisher Scientific; BUV737 anti-CD3 (clone SP34-2; cat. #741872, 5.0 µl), Alexa 700 anti-CD20 (2H7; cat. #560631, 5 µl), BV711 anti-CD4 (clone L200; cat. #563913, 5.0 µl), BV786 anti-CD45 (D058-1283; cat. #563861, 5 µl) from BD Biosciences (San Jose, California, USA) and APC anti-a4b7 (clone A4B7R1; cat. #051514AB, 5.0 µl) from NHP Reagent Resource for 30 minutes at room temperature. Samples were acquired on a BD FACSymphony A5 cytometer and analyzed with FlowJo software 10.6. CD4^+^ T cells were gated as singlets, live cells, CD45^+^ cells, CD3^+^ and CD4^+^ cells.

### 2.13 Luminex analysis in plasma

Plasma collected before vaccination, 2 weeks following the second boost (week 15), and 2 weeks following the third boost (week 42), were analyzed using three MILLIPLEX^®^ Non-Human Primate Multiplex assays (EMD Millipore Corporation, Billerica, MD). The samples were assayed following the manufacturer instructions. Plasma collected before vaccination, week 15 and week 42 were assayed for the following cytokines/chemokines: IL-1β, IL-6, IL-8, IL-10, IL-18, TNF-α, IFN-γ (Cat. #PRCYTOMAG-40K-19). Briefly, samples were thawed on ice, 25 µl of each plasma was loaded singly onto the plate and mixed with 25 µl of assay buffer and 25 µl of magnetic beads. The plates were incubated at 4°C for 18 hours under agitation at 650 rpm. Following the incubation, the plate was washed, 25 µl of detection antibody was added to each well and incubated for 1 h at room temperature (RT). Next, 25 µl of Streptavidin-PE was added to each well and incubated for 30 min at RT. Finally, the plate was washed and 150 µl of sheath fluid was added to each well. Samples were acquired on a Bio-Plex^®^ 200 System (Bio-Rad, Hercules, CA).

### 2.14 Statistical analysis

Statistical analysis was performed using the Wilcoxon signed-rank test or Mann-Whitney test to compare continuous factors between two paired or unpaired groups, respectively. Comparisons of differences between groups in the number of challenges before viral acquisition were assessed using the log-rank (Mantel-Cox) test of the discrete-time proportional hazards model. The average per-risk challenge of viral acquisition was estimated as the total number of observed infections divided by the number of administered challenges. Correlation analyses were performed using parametric Pearson’s correlation or the non-parametric Spearman-rank correlation method with exact permutation and approximate two-tailed p values calculated.

## 3 Results

### 3.1 Design of a V2 α-helical peptide scaffolded on cholera toxin B

**Figure 1:**
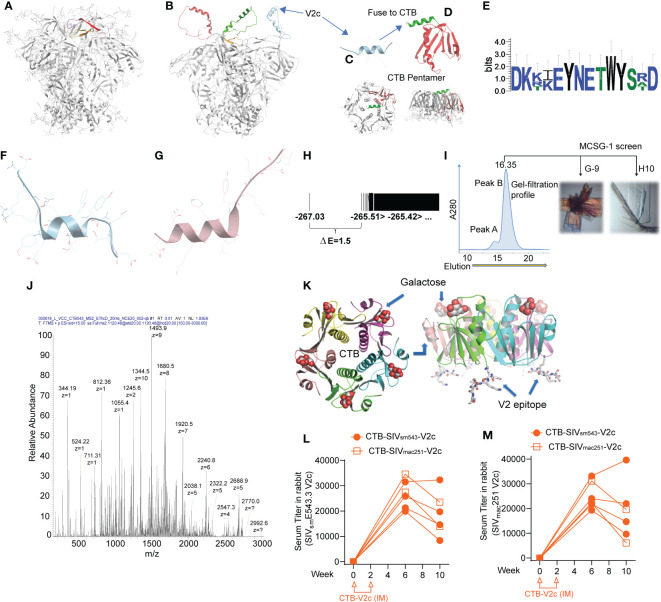
Incorporation of isolated SIV V2 α-helix peptide (V2c) in scaffolded cholera toxin B to generate vaccine. **(A)** Side view of HIV trimeric viral spike structure in complex with apically targeted, broadly neutralizing antibody (PDB 4tvp). V3 loop colored orange, V2b/c epitope colored red. **(B)** Model of HIV viral spike with its V1/V2 domain in the α-helical form exposed at the extreme apex of the viral spike that is observed in its complex with the non-neutralizing mAb CAP228-16H (PDB 6FY1). **(C, D)** Design of CTB-V2c: the isolated V2c peptide fragment fused with the cholera toxin B subunit (red ribbon). Crystallography confirmed that the subunit still formed a stable pentamer, with preserved GM1-ganglioside and mucosal-targeting sites. **(E)** Weblogo of sequence variation in V2c region in SIV_mac251_, SIV_mac239_, SIV_smE660_ and SIV_smE543_. **(F)** Lowest energy conformation from full *ab initio* prediction of V2c from SIV_mac251_. **(G)** V2c from HIV A244 in complex with mAb CH58 (from Liao et al., 2013). **(H)** Energy spectrum of conformations from V2c *ab initio* folding, showing energy gap of 1.5, indicative of a moderately rigid α-helix. **(I)** Gel filtration profile of CTB-V2c-SIV, crystallization screens were conducted using the peak sample and resulted in two crystal forms. **(J)** LC/MS confirming composition of purified CTB-V2c-SIV. **(K)** 2.4 A structural model of CTB-V2c-SIV based on crystallographic electron density from crystal in figure. **(I, L)** Serum V2c-specific IgG titer against SIV_smE543_ V2c probe and **(M)** SIV_mac251_ V2c probe in rabbits vaccinated with CTB-SIV_sm543_-V2c or CTB-SIV_mac251_-V2c. Orange symbols indicate CTB-V2c vaccinated rabbits.

In HIV, the trimeric spike of the virus is capped by the V1/V2 domain, which, in complex with monoclonal antibodies (mAbs) isolated from infected or vaccinated subjects exhibits one of two different conformations. In complex with neutralizing mAbs (e.g. PG9 (49)), V1/V2 conforms to a β-sheet domain (**Figure 1A**). Alternatively, V1/V2 conforms to an α-helical/disordered domain in complex with non-neutralizing mAbs isolated from protected human subjects (CH58 and CAP228) (50, 51), which block viral engagement of the host α_4_β_7_integrin receptor (26) (**Figure 1B**). Mucosal antibodies CH58 and CAP228 target the linear B-cell epitope at the HIV/SIV spike apex and are the antibodies to have been linked to vaccine protection from HIV/SIV acquisition both in humans (33) and in stringent, preclinical non-human primate challenge models (21, 28). An additional study in macaques demonstrated that V1-deleted (ΔV1) SIV envelope immunogens expose the spike-apical V2 region in an α-helical conformation (designated V2c; **Figures 1C, D**) and elicit anti-V2c Abs mediating potent ADCC and correlating with decreased risk of SIV_mac251_ acquisition (27). Significantly, V2c includes peptide positions 164 to 181 (based on HXBC2 strain numbering), which were targeted for immune selection in the human vaccinees of RV144 (25). Further, SIV V2c is recognized by NCI05, a monoclonal antibody cloned from an ALVAC-based vaccinated macaque, P770, which was protected from numerous SIV_mac251_ viral challenges (27). In the same macaque study, additional antibodies were detected recognizing V2b, a peptide with a β-hairpin conformation that binds to mAb NCI09, also obtained from animal P770 (27). In macaques, ADCC mediated by vaccine-elicited antibodies like NCI05 and NCI09 (detected by V2c and V2b in serum, respectively) correlated strongly with decreased risk of virus infection (27, 29, 31). These findings prompted us to design immunogens that could further focus the antibody response to these B-cell epitopes correlated with protection of humans from HIV infection in RV144.

We hypothesized that V2c, in isolation from the rest of the HIV/SIV envelope, could elicit functional, non-neutralizing Abs that contribute to vaccine efficacy. Accordingly, we designed a V2c SIV_smE543_-based peptide with the sequence DKKIEYNETWYSRD and scaffolded it on cholera toxin subunit B (CTB-V2c**)**. V2c structural analysis predicted the peptide would take an α-helical conformation and suggested its potential to be stable *in vivo* (**Figures 1E–H**). We expressed CTB-V2c in *E. coli* and purified it by galactose affinity chromatography to select for stable CTB pentamers, as these are the only CTB forms that bind to galactose and mucosal GM1 gangliosides (52). Moreover, purification of the immunogen also removed endotoxins, which do not bind galactose resin. Gel filtration revealed a pure pentameric fraction of this CTB-V2c chimeric vaccine immunogen. Liquid chromatography-mass spectrometry (LC-MS) confirmed the composition of the molecule, and crystallographic resolution of the structure confirmed a pure, stable pentamer with a dynamic C-terminal peptide insert, consistent with a flexible α-helix DKKIEYNETWYSRD peaked six weeks following the first immunization (**Figures 1I–K**). To assess the immunogenicity of CTB-V2c, 6 rabbits were intramuscularly immunized, two weeks apart, with CTB-SIV_smE543_-V2c or CTB-SIV_mac251_-V2c. Binding antibody titers against SIV_smE543_ peptide DKKIEYNETWYSRD peaked six weeks following the first immunization (**Figure 1L**). Equivalent titers were also observed with the SIV_mac251_ V2c peptide DKTKEYNETWYSTD, differing in three amino acids, in a parallel ELISA assay (**Figure 1M**). Taken together, these data suggested that our CTB-V2c vaccine was successfully immunogenic.

### 3.2 Rhesus macaques vaccinated with 5xCTB-V2c/alum and DA/CTB-V2c/alum generate V2c specific antibody responses

**Figure 2:**
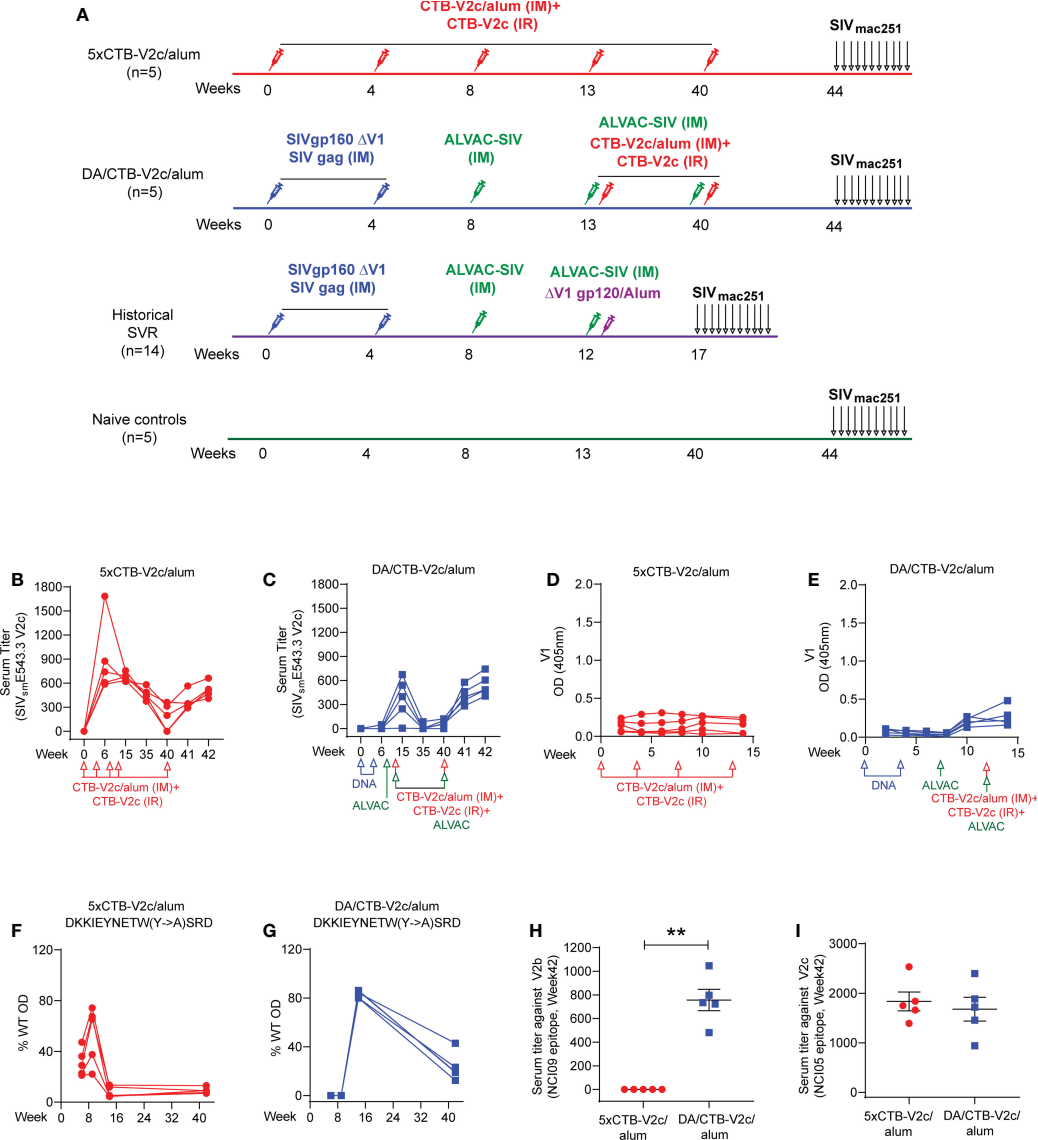
CTB-V2c vaccine induces V2 specific immune responses in macaque model. **(A)** Schematic vaccine study design in macaque model. Fifteen rhesus macaques were subdivided into three groups: 5xCTB-V2c/alum vaccine (n=5), DA/CTB-V2c/alum vaccine (n=5), and controls (n=5). A historical standard vaccine regimen (SVR) was added (n=14) for immune response comparison. Five animals in the 5xCTB-V2c/alum vaccine group were vaccinated with 0.5 mg of CTB-V2c in alum (IM) and 1 mg of CTB-V2c (IR) on weeks 0, 4, 8, 13 and 40. Five animals in the DA/CTB-V2c/alum vaccine group were primed with 2xΔV1DNAgp160+p57 Gag weeks 0, 4; and boosted with ALVAC-SIV encoding *env*, *gag*, and *pol* on week 8 and ALVAC-SIV+ CTB-V2c in alum (IM)+CTB-V2c (IR) on week 13 and 40. Beginning at week 44, protective efficacy against SIV_mac251_ was assessed by subjecting all animals to up to 11 weekly intravaginal viral exposures (arrows) until infection was confirmed. **(B, C)** Anti-V2c serum titers were detected in 5xCTB-V2c/alum and DA/CTB-V2c/alum group. **(D, E)** V1-specific IgG binding in macaques after immunization with the 5xCTB-V2c/alum vaccine or DA/CTB-V2c/alum vaccine. **(F, G)** Percent of serum antibody binding to point mutants in the WT-V2c probe peptide by the two vaccinated group animals. Sequences with mutations indicated displayed above each graph and each line represents one macaque. **(H, I)** Comparison of **s**erum antibody titer against V2b peptide 115 and V2c peptide 150 in 5xCTB-V2c/alum or DA/CTB-V2c/alum vaccinated animals. Red circles indicate 5xCTB-V2c/alum vaccinated macaque group, blue squares indicate DA/CTB-V2c/alum vaccinated macaque group. Data shown in **(H, I)** were analyzed with the Mann-Whitney U test. Horizontal and vertical bars denote mean and SD, respectively. **p < 0.01.

Next, we compared the immunogenicity of the CTB-V2c immunogen to the SVR constituted by 2xΔV1 DNAgp160+p57Gag/1xALVAC-SIV/1xALVAC-SIV+ΔV1gp120/alum boost. In the DA/CTB-V2c/alum regimen, the single ALVAC-SIV/ΔV1gp120 boost was replaced with two ALVAC-SIV/CTB-V2c/alum boosts (2xΔV1DNAgp160+p57Gag/1xALVAC-SIV/2xALVAC-SIV+2xCTB-V2c/alum), whereas the 5xCTB-V2c/alum used only the CTB-V2c/alum immunogen. In the 5xCTB-V2c/alum group five macaques were immunized intramuscularly with 0.5 mg of CTB-V2c formulated in alum simultaneously with topical intrarectal administration of 1 mg of CTB-V2c alone at weeks 0, 4, 8, 13, and 40. In order to observe the behavior of CTB-V2c in the context of the DNA/ALVAC (DA) immunization of the SVR, five animals comprising the DA/CTB-V2c/alum group were immunized intramuscularly with ΔV1gp160 and p57Gag plasmid DNAs (at weeks 0 and 4) and 10^8^ PFU of ALVAC-SIV Gag-pro-gp120TM (vCP2432) at weeks 8, 13, and 40, aligning with the basic protocol of the SVR. Simultaneously with the last two immunizations of vCP2432 (week 13 and 40), CTB-V2c was administered with alum by the muscular route, and without alum by the intrarectal route at the same doses used in the 5xCTB-V2c/alum group. A naïve control group was left unimmunized, and the three immunization groups were compared to assess their immunological parameters (**Figure 2A**).

CTB-V2c delivered as vaccine and as a booster for the ΔV1DNA/ALVAC vaccines elicited equivalent serum ELISA peak titers to the autologous peptide (**Supplementary Figure 1A**) at week 42 in all animals (**Figures 2B, C** **)**. In the 5xCTB-V2c/alum group peak titers against SIV_sm_E543.3 V2c were observed 2 weeks post second immunization with CTB-V2c (week 6), whereas in the DA/CTB-V2c/alum group peak was observed 2 weeks post first immunization with CTB-V2c (week 15; **Figures 2B, C** and **Supplementary Figures 1B, C**). Recall, or the anamnestic response indicative of the establishment of long-term humoral memory, was observed in the rapid antibody rise to peak levels after the 27-week delay between the last two immunizations (**Figures 2B, C**). As expected, a low-level anti-V1 antibody response was observed in the DA/CTB-V2c/alum group, but not in the 5xCTB-V2c/alum group, since the vCP2432 vaccine expresses V1 in a wild-type gp120 (**Figures 2D, E**). Point mutations in the V2c peptide probe revealed that the reactivity of polyclonal sera was dependent on amino acid Y_177_ within the V2 loop (HXBC2 numbering), which is an amino acid conserved between HIV and SIV viruses and is indispensable for CH58 binding to its B-cell epitope (50). Interestingly, anti-V2c sera reactivity, which initially had broader specificity at weeks 6-10 in both groups, focused on Y_177_ by week 15 following four CTB-V2 boosts in the 5xCTB-V2c/alum group, and two CTB-V2 boosts in the DA/CTB-V2c/alum group (**Figures 2F, G** and **Supplementary Figures 1D–G**). Antibody responses to the β-hairpin conformation of V2 were recognized by the NCI09 mAb (V2b; SIV_E543_ peptide 115 _163_GLKRDKTKEYN_173_ (27), in the DA/CTB-V2c/alum group but not the 5xCTB-V2c/alum group (**Figure 2H**). These data demonstrate that antibody responses to V2b can be elicited by the SIV-DA immunizations. Conversely, antibodies to the NCI05-targeted epitope (detected by peptide 150 (53);) were equivalent between the groups (**Figure 2I**). Serum from 5xCTB-V2c/alum vaccinated animals cross-reacted to SIV_mac251_, SIV_smE543_, and SIV_mac239_ V2 loop peptides (**Supplementary Figure 1H**), and plasma from both groups bound to SIV_mac251_ gp120 (**Supplementary Figure 1I**). As expected, the anti-CTB antibody level was higher in the 5xCTB-V2c/alum group compared to the DA/CTB-V2c/alum group at all time points (**Supplementary Figure 1J**).

### 3.3 Differences in functional antibody and immune cell responses in the three vaccine regimens

**Figure 3:**
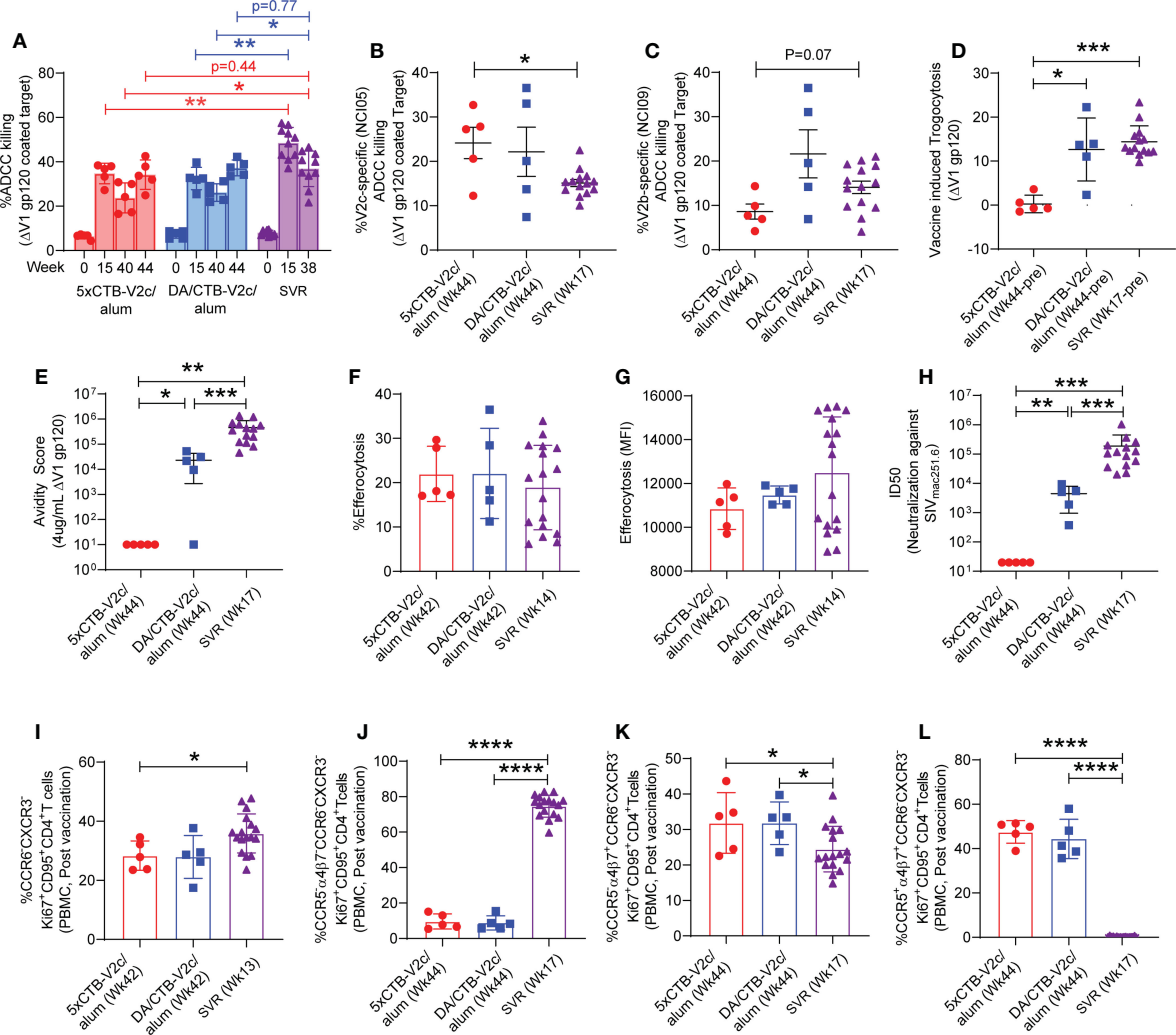
Comparison of non-neutralizing antibody, neutralizing antibody, efferocytosis and Th2 cell response among different vaccine group of macaques. **(A)** Longitudinal percent ADCC killing in animals vaccinated with different vaccine regimens. **(B, C)** Comparison of V2-specific ADCC killing among 3 groups of vaccinated animals before challenging with SIV_mac251_. Comparison of **(D)** Trogocytosis against ΔV1 gp120, **(E)** avidity score against ΔV1 gp120, **(F)** percentage efferocytosis, **(G)** Efferocytosis MFI, **(H)** Neutralizing antibody, **(I)** Th2 cells, **(J)** CCR5^-^ α_4_β_7_- Th2 cells, **(K)** CCR5^-^ α_4_β_7_^+^ Th2 cells, and **(L)** CCR5^+^ α_4_β_7_^+^ Th2 cells among 5xCTB-V2c/alum, DA/CTB-V2c/alum and SVR vaccinated animals. Data shown in **(A–L)** were analyzed with the Mann-Whitney U test or Wilcoxon signed-rank test. Horizontal and vertical bars denote mean and SD, respectively. Red circles indicate 5xCTB-V2c/alum vaccinated macaque group, blue squares indicate DA/CTB-V2c/alum vaccinated macaque group, purple triangles indicate SVR group (2xDNA, 1xALVAC-SIV, 1xALVAC-SIV +ΔV1 gp120/alum boost). *p < 0.05, **p < 0.01, ***p < 0.001, ****p < 0.0001.

The functionality of plasma anti-SIV antibodies was determined by ADCC assays, as it has been repeatedly reproduced as a correlate of decreased risk of SIV_mac251_ acquisition in the SVR (27, 29, 31). Using human PBMCs as effector cells and CEM cells coated with monomeric ΔV1 gp120 protein as target cells, we observed no difference in the overall kinetic of ADCC activity following immunization of animals in the 5xCTB-V2c/alum or DA/CTB-V2c/alum groups compared to the standard vaccination protocol (**Figure 3A**). ADCC killing was equivalent in both the 5xCTB-V2c/alum and DA/CTB-V2c/alum groups following the last immunization (week 44), but it was significantly lower at weeks 40 and 44 than it was at week 38 in a subset of unchallenged animals in the SVR, which did not include the CTB-V2c boost (**Figure 3A**). This data demonstrated higher ADCC in the standard protocol at more than six months (26 weeks) after the last immunization.

Next, we analyzed V2-specific ADCC using NCI05 or NCI09 F(ab’)2 to specifically inhibit ADCC directed to V2c and V2b, respectively. V2c-specific ADCC revealed by NCI05 F(ab’)2 was equivalent in the 5xCTB-V2c/alum and DA/CTB-V2c/alum groups and significantly higher than in the SVR (**Figure 3B**). In contrast, V2b-specific ADCC revealed by NCI09 F(ab’)2 was lower in the 5xCTB-V2c/alum group (**Figure 3C**) as expected, since the V2b epitope was not included in the 5xCTB-V2c/alum vaccine regimen and the ADCC response could therefore only be elicited from the DNA/ALVAC combination in the DA/CTB-V2c/alum group.

Although trogocytosis by anti-HIV antibodies is common (54), the role of antibody-dependent trogocytosis in SIV infection is not clear. We measured trogocytosis exclusively, since it may overlap with ADCC, and observed a lower level of activity in the 5xCTB-V2c/alum group compared to the other two vaccine regimens (**Figure 3D**). We also observed a high serum titer against the V2c region in the 5xCTB-V2c/alum group (**Figure 2B**) and were therefore interested in the overall avidity of the antibody towards the ΔV1gp120 protein used for immunization. Here, the overall antibody avidity was higher in the SVR compared to the two other animal groups (**Figure 3E**). We next tested antibody independent CD14^+^ cell efferocytosis, which has been reported to correlate with a decreased risk of SIV_mac251_ acquisition (29, 31). The percentage of CD14^+^-engulfing neutrophils in the efferocytosis assay or the extent of engulfment (MFI) in CD14^+^ cells did not differ in the three groups (**Figures 3F, G**).

Analysis of neutralizing antibody titers demonstrated that none of the three vaccine regimens elicited neutralizing antibodies against the challenge virus (data not shown). A higher neutralizing antibody response was observed against the tier 1 SIV_mac251.6_ in the SVR compared to the other 2 vaccine regimens. The 5xCTB-V2c/alum group showed no neutralizing antibody responses (**Figure 3H**), suggesting, as suspected, that V2c is not a neutralization epitope. This data is the first precise evaluation of a vaccine-elicited non-neutralizing immune response to the viral spike apex not influenced by the presence of neutralizing antibodies.

We next turned our attention to changes in the CCR5 profile. Gut-homing CCR5^-^ CD4^+^ Th2 cells have been associated with decreased risk of SIV_mac251_ acquisition (28). Furthermore, CCR5^+^ α_4_β_7_^+^Ki67^+^ CD4^+^ T cells are more susceptible to HIV infection compared to CCR5^-^ α_4_β_7_^-^Ki67^+^ CD4^+^ T cells (55). Both 5xCTB-V2c/alum and DA/CTB-V2c/alum vaccinated groups showed comparable levels of vaccine induced Th2 cells, CCR5^-^ α_4_β_7_^-^ Th2 cells, CCR5^-^ α_4_β_7_^+^ Th2 cells, and CCR5^+^ α_4_β_7_^+^ Th2 cells (**Figures 3I–L**). Interestingly, the frequency of Th2 cells in the Ki67^+^CD95^+^CD4^+^ T cell subset and the frequency of CCR5^-^ α_4_β_7_^-^ Th2 cells in the CCR6^-^ CXCR3^-^Ki67^+^CD95^+^CD4^+^ subset were higher in the SVR than in the 5xCTB-V2c/alum and DA/CTB-V2c/alum groups (**Figures 3I, J**). In contrast, the frequency of α_4_β_7_^+^ Th2 cells was higher in the 5xCTB-V2c/alum and the DA/CTB-V2c/alum groups (**Figures 3K, L**). Like Th2, the Th1 and Th17 cell subsets were comparable in the 5xCTB-V2c/alum and DA/CTB-V2c/alum vaccinated animals (**Supplementary Figures 2A–H**). While CCR5^-^ α_4_β_7_^-^ Th1 cells were higher in the SVR (**Supplementary Figure 2B**), all other Th1 subsets were lower in the SVR compared to the 5xCTB-V2c/alum and DA/CTB-V2c/alum groups (**Supplementary Figures 2A, C, D**). However, CCR5^+^ α_4_β_7_^+^ Th17 cells were lower in the SRV (**Supplementary Figure 2H**), and all other Th17 cell subsets were higher in SRV than in the other two vaccine regimens (**Supplementary Figures 2E–G**). Taken together, these data suggest that the SVR generates a higher frequency of infection resistant CD4^+^ T cells than do the other two regimens.

Analysis of cytokines in plasma following vaccination demonstrated that plasma levels of IFN-γ, IL-10, IL-1β, IL-6, IL-8, TNFα, and IL-18 were comparable in animals vaccinated with both the 5xCTB-V2c and DA/CTB-V2c/alum regimens (**Supplementary Figure 3**). A similar analysis was not done in the SVR.

### 3.4 Correlates of delayed SIV_mac251_ acquisition

**Figure 4:**
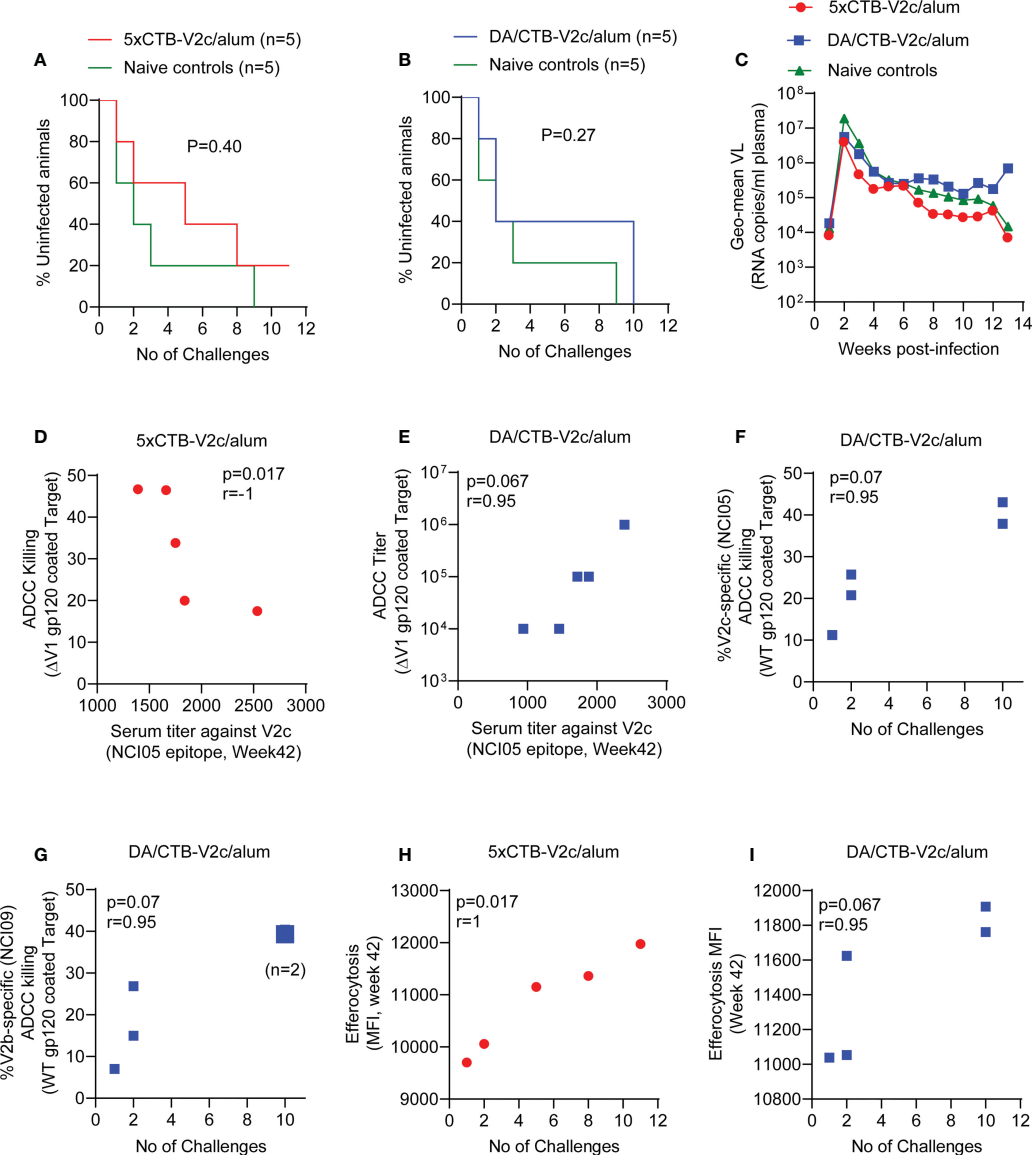
Infection rate, SIV plasma virus, and association of ADCC and efferocytosis responses with SIV acquisition. **(A)** Comparison of SIV acquisition in 5xCTB-V2c/alum vaccinated (n=5) and **(B)** DA/CTB-V2c/alum vaccinated macaques (n=5) compared to naïve control group (n=5) animals. **(C)** VL geometric means of all macaque groups over time. **(D)** Correlation of ADCC killing in the 5xCTB-V2c/alum vaccinated group and **(E)** ADCC titer in DA/CTB-V2c/alum vaccinated group with respective V2c specific serum antibody titer. **(F, G)** Correlation of V2-specific ADCC killing with number of rectal SIV_mac251_ challenges required to infect the animals in DA/CTB-V2c/alum vaccinated group. Correlation of efferocytosis MFI in **(H)** the 5xCTB-V2c/alum and **(I)** in DA/CTB-V2c/alum vaccinated group with number of intra-rectal challenges. Data shown in **(A, B)** were analyzed with log-rank (Mantel–Cox) test. Data shown in **(D–I)** were analyzed with the Spearman correlation test. Red circles indicate 5xCTB-V2c/alum vaccinated macaque group, blue squares indicate DA/CTB-V2c/alum vaccinated macaque group.

The limited number of animals immunized in the 5xCTB-V2c/alum and DA/CTB-V2c/alum groups precluded us from determining a statistically significant decrease in the risk of SIV_mac251_ acquisition compared to unvaccinated controls. Nevertheless, we analyzed per-challenge risk of virus acquisition in each vaccinated group following 11 low-doses of the same SIV_mac251_ virus stock previously used in the standard vaccine regimen. Five animals were simultaneously exposed to SIV_mac251_ for virus infectivity as a control group. The acquisition curve did not significantly differ in the control group compared to the 5xCTB-V2c/alum and DA/CTB-V2c/alum groups (**Figures 4A, B**). As previously published, the SVR has a vaccine efficacy of 57% (p=0.04) (27). In contrast, plasma viral RNA levels following infection did not differ among the 5xCTB-V2c/alum, DA/CTB-V2c/alum and control groups (**Figure 4C**).

Immune correlate data for the SVR were not included in this study, since they have been published previously (27–29). Our analyses of immune correlates and of time to virus acquisition in the 5xCTB-V2c/alum and DA/CTB-V2c/alum groups demonstrated a surprisingly strong inverse correlation between ADCC and serum reactivity to peptide 150 (V2c) in the 5xCTB-V2c/alum regimen (**Figure 4D**). In contrast, in the DA/CTB-V2c/alum group there was a strong trend for a positive correlation of delayed viral acquisition with higher ADCC titers (**Figure 4E**). A similar trend of correlation with protection was observed in the DA/CTB-V2c/alum group for V2 (NCI05 and NCI09) specific ADCC (**Figures 4F, G** **)**, suggesting both antibody targets contribute to delayed acquisition. Efferocytosis is a mechanism by which CD14^+^ cells remove apoptotic cells and help maintain inflammation. As in the SVR (29, 31), increased efferocytosis strongly correlated with delayed SIV_mac251_ acquisition in both 5xCTB-V2c/alum and DA/CTB-V2c/alum groups (**Figures 4H, I**).

**Figure 5:**
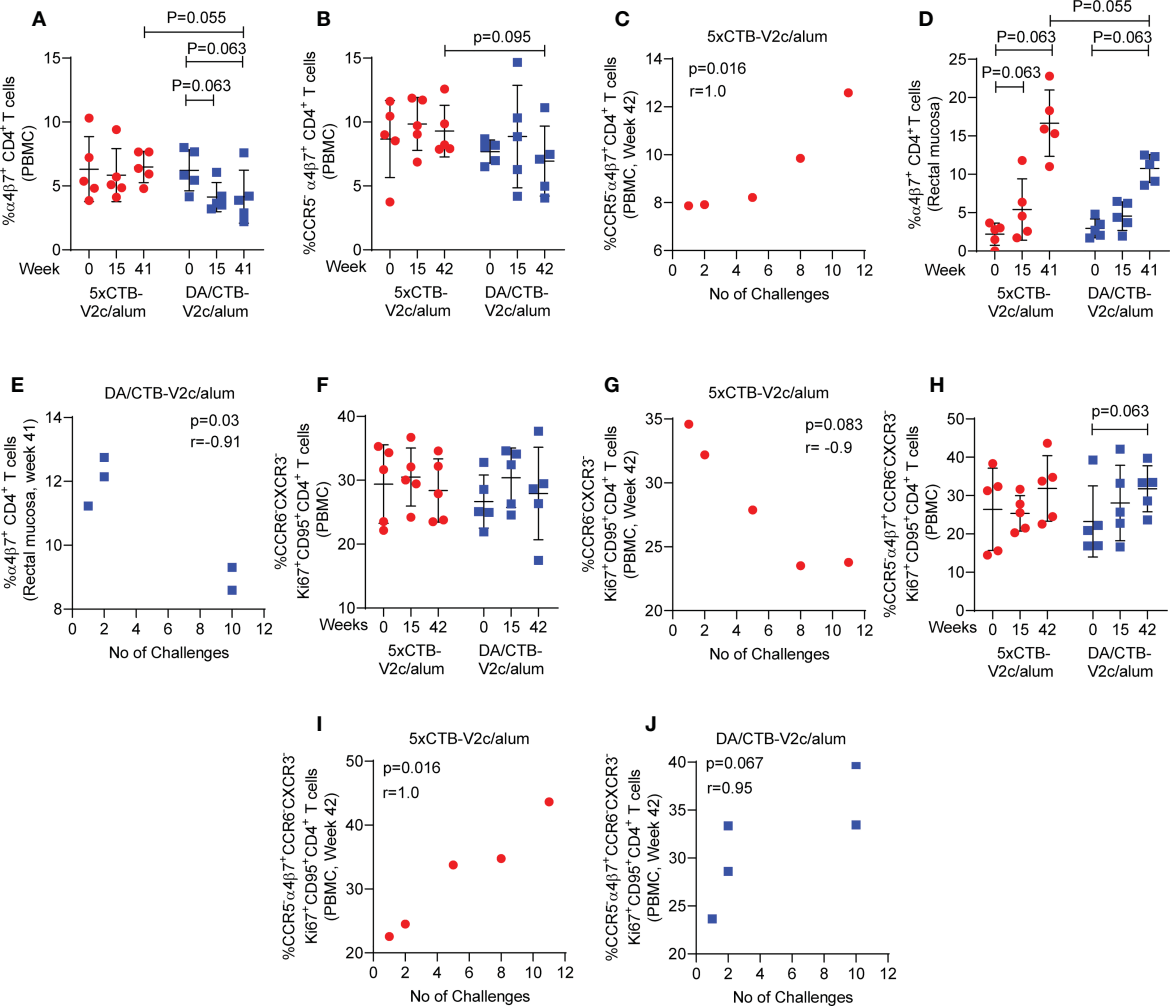
Comparison of different T cell subsets and their association with SIV acquisition. Comparison of the frequency of circulating **(A)** α_4_β_7_^+^ CD4^+^ T cells and **(B)** CCR5^-^ α_4_β_7_^+^ CD4^+^ T cells over the course of vaccination. **(C)** Correlation of circulating CCR5^-^ α_4_β_7_^+^ CD4^+^ T cells with number of challenges in the 5xCTB-V2c/alum group. **(D)** Comparison of the frequency of mucosal α_4_β_7_^+^ CD4^+^ T cells over the course of vaccination. **(E)** Correlation of mucosal α_4_β_7_^+^ CD4^+^ T cells with number of challenges in DA/CTB-V2c/alum group. **(F)** Comparison of the frequency of circulating memory Th2 cells over the course of vaccination. **(G)** Correlation of circulating memory Th2 cells with number of challenges in 5xCTB-V2c/alum group. **(H)** Comparison of the frequency of circulating memory CCR5^-^ α_4_β_7_^+^ Th2 cells over the course of vaccination. Correlation of circulating memory CCR5^-^ α_4_β_7_^+^ Th2 cells with number of challenges in **(I)** 5xCTB-V2c/alum group and **(J)** in DA/CTB-V2c/alum group. Data shown in **(A, B, D, F, H)** were analyzed Mann-Whitney U test or Wilcoxon signed-rank test. Horizontal and vertical bars denote mean and SD, respectively. Data shown in **(C, G, I, J)** were analyzed with the Spearman correlation test. Data shown in **(E)** were analyzed with the Pearson’s correlation test. Red circles indicate 5xCTB-V2c/alum vaccinated macaque group, and blue squares indicate DA/CTB-V2c/alum vaccinated macaque group.

Post vaccination (week 42), circulating α_4_β_7_^+^ CD4^+^ T-cells (**Figure 5A**) and CCR5^-^ α_4_β_7_^+^ CD4^+^ T-cells (**Figure 5B**) showed a higher trend of frequency in the 5xCTB-V2c/alum group compared to the DA/CTB-V2c/alum group. Interestingly, circulating CCR5^-^ α_4_β_7_^+^CD4^+^ T-cells correlated with delayed SIV_mac251_ acquisition in the 5xCTB-V2c/alum group (**Figure 5C**). On the other hand, post vaccination rectal α_4_β_7_^+^ CD4^+^ T-cells were increased in both the 5xCTB-V2c/alum and DA/CTB-V2c/alum groups (**Figure 5D**) and were associated with increased SIV_mac251_ acquisition in the latter (**Figure 5E**). Vaccination with 5xCTB-V2c/alum or DA/CTB-V2c/alum did not change the frequency of Th2 memory cells in blood (**Figure 5F**), but a trend of blood Th2 cells and accelerated SIV_mac251_ acquisition was observed in the 5xCTB-V2c/alum group (**Figure 5G**). The CCR5^-^ α_4_β_7_^+^ subset of Th2 memory cells were comparable in the 5xCTB-V2c/alum and DA/CTB-V2c/alum vaccinated groups (**Figure 5H**). The CCR5^-^ α_4_β_7_^+^ subset specifically correlated with delayed acquisition in 5xCTB-V2c/alum (**Figure 5I**) and a trend of protection in the DA/CTB-V2c/alum group (**Figure 5J**), further suggesting the importance of circulating CCR5^-^ α_4_β_7_^+^ CD4^+^ T-cells in delaying SIV acquisition.

## 4 Discussion

Post-clinical analysis of RV144 and the large number of follow-up studies have clearly identified the major correlates of protection afforded by the DNA-ALVAC vaccine platform. These studies have established strong evidence of a potential correlation between the non-neutralizing antibodies that target B-cell epitopes in the V2 loop at the apex of the viral spike and protection from HIV/SIV viral acquisition (20). Antibody targeting of these epitopes is likely necessary for protection against HIV/SIV infection, however *in vivo* administration of non-neutralizing antibodies in macaques has also shown that they are not on their own sufficient to mediate protection (53). The continuous progress made in dissecting the correlates of protection in RV144 moreover offers insight into the stark contrast between this clinical trial’s measured success and the lack of protection in the follow-up HVTN trial (16). The primary differences between RV144 and HVTN702 were their use of different circulating viruses (clade A vs clade C), different host populations (Thai vs Southern African), and the choice of vaccine adjuvant (alum vs MF59). Our studies in macaques have mirrored the diverging results of these human trials and suggest that the adjuvant in particular may have produced profoundly different antibody functions and cellular immune responses, which likely account for the lack of protection in the latter trial. The available evidence therefore suggests that the most promising course of action is to continue to improve on the DNA-ALVAC platform by honing in on its known correlates of protection.

In macaque studies, it has been shown that the presence of mucosal NKp44^+^IL17^+^ cells (21, 28, 30), efferocytosis (29, 31), and Th1/Th2 cells expressing no or low levels of CCR5 (27, 28, 31) and ADCC activity (27, 29, 31) are important components to protect against SIV infection. Here, we more precisely dissected some of the accompanying immune responses responsible for protection from viral acquisition by focusing our attention on V2c, holding the vaccine-elicited antibodies constant and selectively altering the surrounding polyclonal Abs, adjuvant, and viral/DNA primes.

We found that more than one Ab target at the apex may be required in a vaccine for efficacious Ab avidity against gp120, ADCC, and trogocytosis, even in a setting of desirable vaccine-elicited Abs and protective circulating T-cell responses. The DA primed animals as well as the SVR animals (27) both showed correlations of V2b/NCI09 and V2c/NCI05 specific ADCC activity with delayed viral acquisition. No such correlation was observed for the 5xCTB-V2c/alum vaccine, which only elicited Abs to V2c, as the vaccine only contained the V2c epitope. Considering the difference in avidity results (**Figure 3E**), it is possible that Ab targeting of more than one location on the viral spike apex is required for Abs to cross-link spikes to achieve avidity.

The low ADCC and trogocytosis response in the presence of low avidity against gp120 observed in the 5xCTB-V2/alum group suggested that higher avidity against gp120 might enhance these immune responses. The negative correlation observed between ADCC and V2c titer in the same group (**Figure 4D**) moreover suggests that a high level of vaccine elicited epitope-focused Abs could compete with virion-bound Abs that mediate ADCC and generate a “prozone-like” effect (56). Epitope-focused vaccine and mAb approaches may benefit from optimization of the Ab titer and/or combinations of epitopes.

5xCTB-V2/alum vaccination does not change the frequency of circulating α_4_β_7_^+^ CD4^+^ T-cells (**Figure 5A**) but increased the frequency of rectal mucosal α_4_β_7_^+^ CD4^+^ T-cells (**Figure 5D**). These cells might act as targets of HIV/SIV, since the V2 loop binds host α_4_β_7_. Since CTB mucosal vaccination elevates MaDCAM-1 in the gut mucosa (57), eliminating the mucosal immunization of the 5xCTB-V2c/alum vaccine regimen might improve the vaccine outcome. In this process, mucosal anti-V2c antibodies might be sacrificed for the benefit of having fewer α_4_β_7_^+^ targets in the mucosa.

HIV expresses decoy or enhancing epitopes to evade immune responses while suppressing or immuno-attenuating sites of vulnerability (58). HIV exhibits an abundance of antigenic decoys at the apex of its viral spike, which help the virus to escape from protective neutralizing or non-neutralizing antibody responses. We previously demonstrated that removal of the V2c-masking, V1 loop region from the apex of the viral spike improves vaccine efficacy (27), suggesting that immunodominant V1 loops act as decoys for the immune system. We have also demonstrated that V2-specific immune responses play an important role in providing protection against SIV acquisition (27). Here, we engineered epitope-focused immunogens to amplify the immunogenicity of suppressed V2c epitopes, which are a demonstrated correlate of protection in RV144 (59). Notably, the V2c epitope (also termed “V2i” in the literature (60)) is a target of mAbs CH58 and CAP228, as shown by crystallography. In our CTB-V2c/alum vaccine, V2c showed an α-helical conformation at the spike apex of gp120 (**Figure 1A**). Indeed, the polyclonal serum Abs elicited by our V2c design were dependent on the V2 loop amino acid Y177 (**Figures 2H, I** **)**, which suggests a focused elicitation *in vivo* of CH58/CAP228-like, non-neutralizing antibodies in isolation from any other distracting, anti-HIV/SIV Abs. Prior efforts at focusing HIV/SIV vaccine immunogens to individual B-cell epitopes failed to elicit Abs in non-human primates (61). These efforts also failed to elicit functional serum anti-HIV/SIV Abs in mammals (62–65), as well as failing to elicit any detectable anti-viral mucosal Abs. Conversely, antibody targeting of scaffolded V1/V2 domains has been investigated with some promising results, especially with respect to the magnitude and cross-virus breadth of the resulting Abs (56, 66). Here, we achieved an anamnestic response with both the 5xCTB-V2c/alum and DA/CTB-V2c/alum groups, further suggesting the utility of CTB as a scaffold-adjuvant, as long as more than one apical epitope is included, and mucosal immunization is avoided.

Our prior work with the V3 loop remains the only study that elicited, by design, a neutralizing, polyclonal serum Ab response in mammals that recapitulated the neutralizing specificity of the monoclonal Ab which served as the basis for the design of this focused immunogen (37). Based on recent successes with COVID vaccines (67, 68), focused elicitation of Abs targeting broadly conserved, protection-associated individual epitopes by vaccination might be the basis of universal HIV or COVID vaccines, regardless of the delivery platform (mRNA, DNA, protein, etc.). Notably, ACE receptor and protective neutralizing anti-SARS-CoV-2 Abs target the extreme apex of the viral spike at its central axis, equivalent to the NCI09/V2b epitope in HIV/SIV, while secondary neutralizing epitopes are located at the apex periphery on the N-terminal domain of SARS-CoV-2, equivalent to NCI05/V2c (69). Though speculative, it is intriguing to consider that these two locations might be important sites of vulnerability in all Class I fusion viruses, including SARS-Cov-2, RSV, and many others.

Overall, our SVR regimen, which inspired the vaccines in this study, generated higher levels of ADCC responses (**Figure 3A**), however, the 5xCTB-V2c vaccine regimen generated a higher level of V2c- and a lower level of V2b-specific ADCC compared to the SVR (**Figures 3B, C** **)**. Moreover, trogocytosis and avidity against gp120 were lower in animals vaccinated with 5xCTB-V2c/alum, and neutralizing antibodies were absent entirely (**Figures 3D–F**). Efferocytosis on the other hand was comparable among all three immunization groups (**Figures 3G, H** **)**. Notably, the frequency of CCR5^-^ α_4_β_7_^-^ Th2, Th1 and Th17 cells was higher in animals immunized with the SVR (**Figure 3J** and **Supplementary Figures 2B, F**). Coordinated cellular immune responses thus appear to be sensitive to the DNA/ALVAC/alum prime/boost, which suggests that heterologous priming and/or adjuvants can profoundly enhance protection conferred by apex-focused, vaccine-elicited antibodies. Specifically, this study provides further evidence that priming the immune system with virus-like particles formed by the co-administration of gp160 and p57 DNA vaccines and increasing the production of VLPs by boosting with ALVAC-SIV, may be important to generate protective antibody functions elicited by these vaccines. The CTB-V2c immunogen in the 5xCTB-V2c and in DA/CTB-V2c/alum vaccine regimens showed distinct immune responses compared to the protective SVR, which might have been the cause for the failure of CTB-V2c based immunization in this specific set of vaccine regimens. However, the number of animals was too small to confirm futility of the CTB-V2c based vaccine regimen. Future vaccine studies focusing on the generation of V2c and/or V2b responses with the suitable route of immunization as well as administration of specific DNA or viral vector prime, and/or adjuvants boosts might generate desirable HIV vaccine efficacy.

## Data availability statement

The raw data supporting the conclusions of this article will be made available, if possible and appropriate, upon request to the corresponding authors.

## Ethics statement

Rabbit work was approved by the Pocono Rabbit Farm and Laboratory IACUC, with umbrella approval by the NYU Langone Health IACUC committee. Macaque work was approved by the Tulane National Primate Research Center, Covington, LA and the NCI Animal Care and Use Committee (ACUC) prior to study initiation.

## Author contributions

TC conceived the study in consultation with GF. MAR, GF, and TC wrote the paper. MAR performed ADCC assays, V2-specific ADCC assays, flow cytometry assay, cellular immune assays, efferocytosis assay, analyzed the data, and prepared figures. TC designed the immunogens and diagnostic peptides. MB-F performed confirmatory molecular modeling and serological assays. YP expressed and characterized the immunogens, including crystallography. ISdC and MB coordinated standard vaccine regimen study. ISdC performed serum gp120 binding assay and generated F(ab’)2 fragments for ADCC assay. MB performed efferocytosis assay. SB and MR performed Surface Plasmon Resonance (Biacore) assay. XS, LW, and GT designed and contributed BAMA assay. XS and CL contributed neutralization assays. SS performed Luminex assay. KN’E and DP-P performed trogocytosis assay. PA and RV coordinated and performed the macaque studies. All authors contributed to the article and approved the submitted version.
