# Supplementary material for: Cholera toxin B scaffolded, focused SIV V2 epitope elicits antibodies that influence the risk of SIVmac251 acquisition in macaques
Source: Front Immunol. 2023 Apr 21;14:1139402. doi: 10.3389/fimmu.2023.1139402 (PMC10160393; doi:10.3389/fimmu.2023.1139402)
Supplement: Supplementary file 1 [file DataSheet_1.docx]

Supplementary Material

Cholera toxin B scaffolded, focused SIV V2 epitope elicits antibodies that influence the risk of SIV_mac251_ acquisition in macaques

Mohammad Arif Rahman^1^, Manuel Becerra-Flores^2^, Yury Patskovsky^2^, Isabela Silva de Castro^1^, Massimiliano Bissa^1^, Shraddha Basu^3,6^, Xiaoying Shen^4^, LaTonya D. Williams^5^, Sarkis Sarkis^1^, Kombo F. N’guessan^3,6^, Celia LaBranche^4^, Georgia D. Tomaras^5^, Pyone Pyone Aye^7^, Ronald Veazey^8^, Dominic Paquin-Proulx ^3,6^, Mangala Rao^3^, Genoveffa Franchini^1*^, Timothy Cardozo^2*^

*** Correspondence:** Tiomothy Cardozo: [timothy.cardozo@nyulangone.org](mailto:timothy.cardozo@nyulangone.org); Genoveffa Franchini: franchig@mail.nih.gov


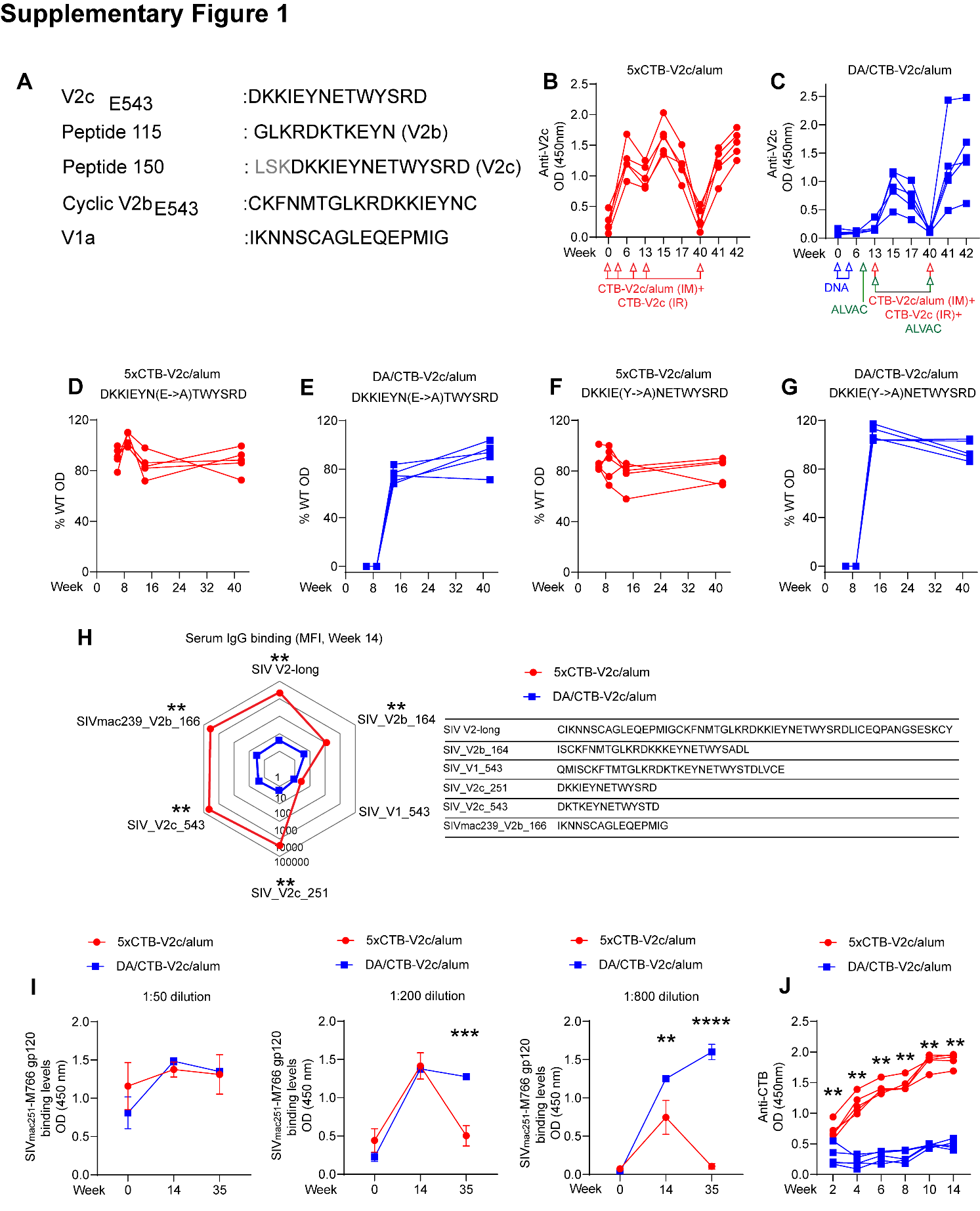


**Supplementary Figure 1.** **Serum antibody responses based on sequence of different peptides**. **(A)** V2c E543 linear peptide, V2b 115 linear peptide, V2c 150 linear peptide, cyclic V2c E543 peptide and V1a peptide sequences are noted. **(B, C)** Serum SIVsmE543 V2c-specific IgG binding in macaques after immunization with 5xCTB-V2c/alum vaccine or with DA/CTB-V2c/alum vaccine. **(D-G)** Percent of serum antibody binding to point mutants in the WT-V2c probe peptide by 5xCTB-V2c/alum vaccinated group or DA/CTB-V2c/alum vaccinated macaque group animals. Sequences with mutations indicated displayed above each graph and each line represents one macaque. **(H)** MFI of serum IgG binding to various V1/V2 antigens at week 14. The radar plot represents the mean value of serum IgG binding. **(I)** SIV_mac251-M766_ gp120 IgG antibody binding levels were measured in the serum at 1:50, 1:200 and 1:800 dilutions. The data plotted was subtracted from the blank and is represented as mean ± SD. **(J)** Serum CTB-specific IgG binding in macaques after immunization with 5xCTB-V2c/alum vaccine or with DA/CTB-V2c/alum vaccine. Data shown in **(H, J**) were analyzed with Mann-Whitney U test. Data shown in **(I**) were analyzed with 2way ANOVA multiple comparisons, with Sidak's multiple comparisons test were used to compare the difference in the OD mean in each time point between the groups. Red circles indicate 5xCTB-V2c/alum vaccinated macaque group, blue squares indicate DA/CTB-V2c/alum vaccinated macaque group. **p < 0.01, ***p < 0.001, ****p < 0.0001.


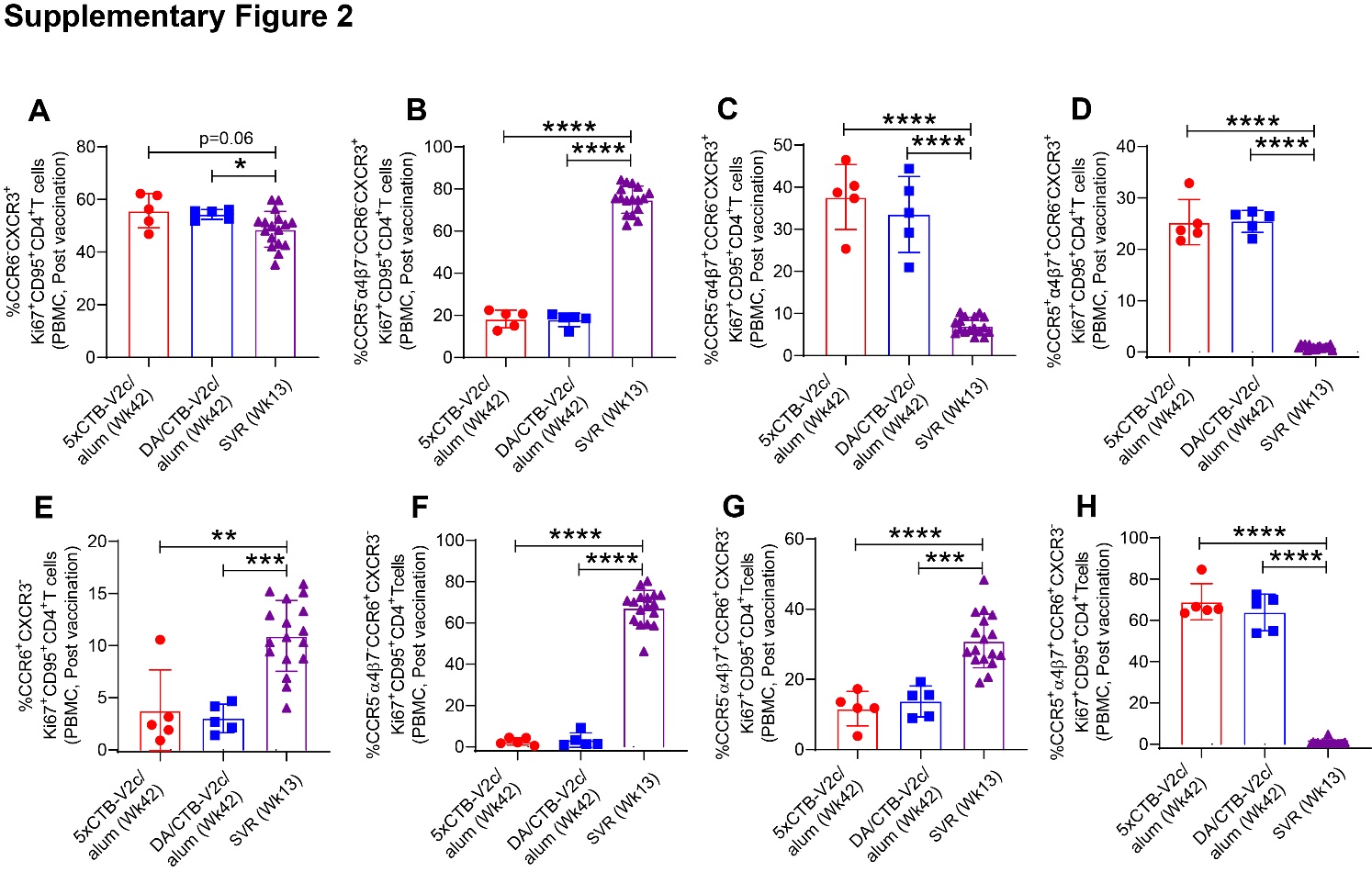
**Supplementary Figure 2.** **Comparison of Th1 and Th17 cell response among different vaccine** **group of macaques.** Comparison of **(A)** Th1 cells, **(B)** CCR5-α4β7- Th1 cells, **(C)** CCR5- α4β7+ Th1 cells, **(D**) CCR5+ α4β7+ Th1 cells, **(E)** Th17 cells, **(F)** CCR5-α4β7- Th17 cells, **(G)** CCR5- α4β7+ Th17 cells, and **(H**) CCR5+ α4β7+ Th17 cells among 5xCTB-V2c/alum, DA/CTB-V2c/alum and SVR vaccinated animals. Data shown in **(A-H)** were analyzed with the Mann-Whitney U test. Horizontal and vertical bars denote mean and SD, respectively. Red circles indicate 5xCTB-V2c/alum vaccinated macaque group, blue squares indicate DA/CTB-V2c/alum vaccinated macaque group, purple triangles indicate SVR group (2xDNA, 1xALVAC-SIV, 1xALVAC-SIV +ΔV1 gp120/alum boost). *p < 0.05, **p < 0.01, ***p < 0.001, ****p < 0.0001.


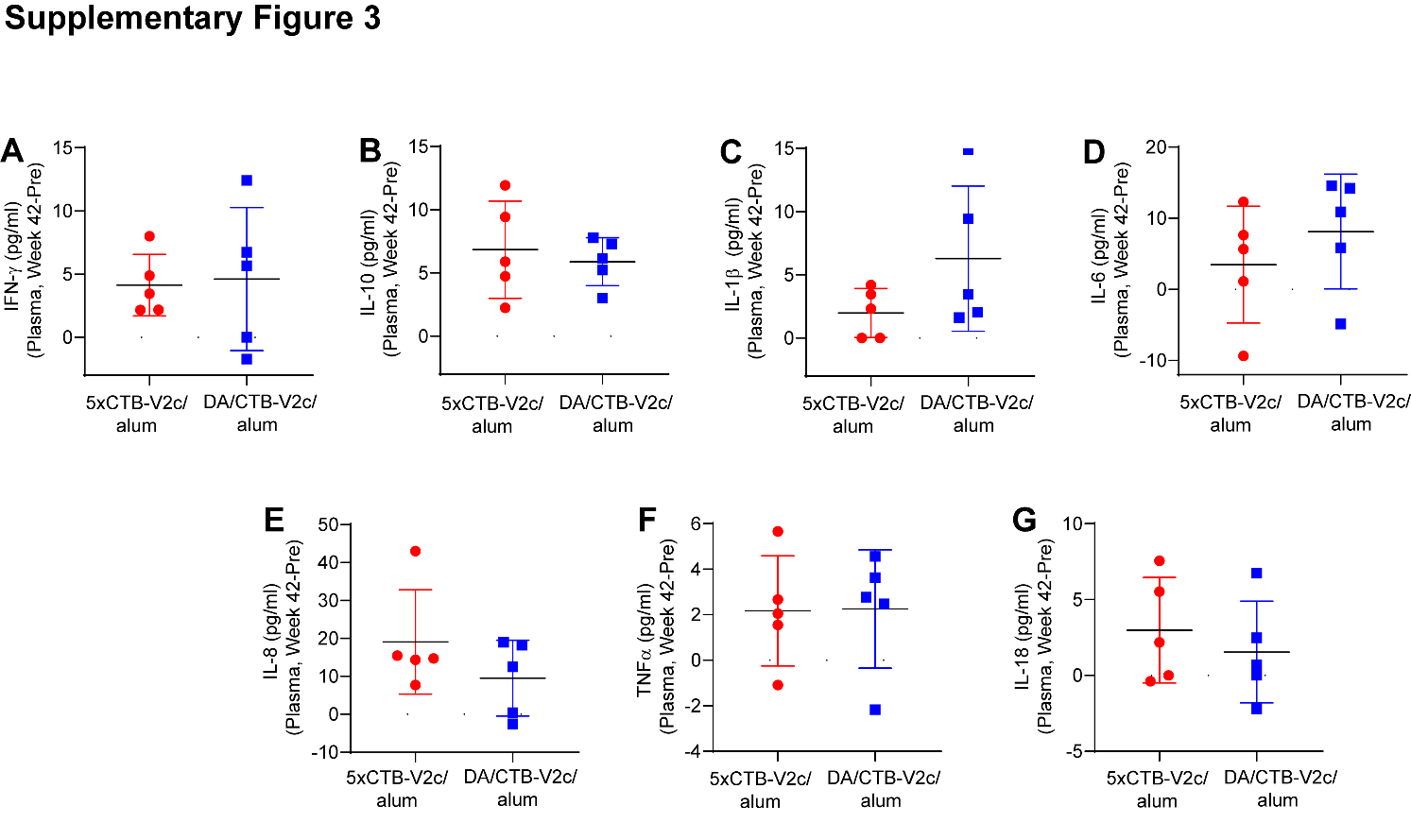
**Supplementary Figure 3: The 5xCTB-V2c/alum vaccinated and DA/CTB-V2c/alum vaccinated animal group shows comparable vaccine induced cytokine responses.** Comparison of **(A)** IFN-g, **(B)** IL-10, **(C)** IL-1β, **(D)** IL-6, **(E)** IL-8, **(F)** TNFα and **(G)** IL-18 between CTB-V2c and ΔV1 DNA/ALVAC/CTB-V2c vaccinated animals. Data shown in (**A-G**) were analyzed with the Mann-Whitney U test. Horizontal and vertical bars denote mean and SD, respectively. Red circles indicate 5xCTB-V2c/alum vaccinated macaque group, blue squares indicate DA/CTB-V2c/alum vaccinated macaque group.
